# Developing a predictive nomogram for AMI in elderly patients with AHF: a retrospective analysis

**DOI:** 10.3389/fmed.2025.1555596

**Published:** 2025-07-09

**Authors:** Qili Yu, Tingting Song, Rui Cui, Li Liu

**Affiliations:** Department of Cardiology, The First Hospital of Qinhuangdao, Qinhuangdao, Hebei, China

**Keywords:** acute myocardial infarction, acute heart failure, elderly, nomogram, prediction model

## Abstract

**Background:**

This study focuses on the clinical issue of acute myocardial infarction (AMI) in the context of acute heart failure (AHF), particularly among the elderly population. Elderly patients with AHF experiencing AMI represent a severe cardiac condition with poor prognosis. Hence, this research aims to analyze potential risk factors and establish a clinical prediction model using logistic regression to facilitate early assessment and guide clinical decisions.

**Methods:**

A retrospective analysis design was employed, selecting elderly AHF patients hospitalized in the Cardiovascular Department of Qinhuangdao City First Hospital from October 2019 to December 2023. Patient history and clinical data were analyzed using LASSO regression and logistic regression to identify and analyze predictors of AMI, leading to the construction of a nomogram. The model’s predictive performance was evaluated using the concordance index, receiver operating characteristic curve, decision curve analysis, and clinical impact curves to gain insights into the nomogram’s accuracy and clinical utility.

**Results:**

The study included 1,904 patients. Logistic regression analysis identified age, coronary heart disease, diabetes, pulmonary infection, ventricular arrhythmia, hyperlipidemia, hypoalbuminemia, left ventricular diastolic diameter (LVDD), and left ventricular ejection fraction (LVEF) as independent risk factors for AMI during hospitalization. The predictive model was formulated as follows: Logit(P) = −7.286 + 0.065 × Age + 0.380 × Coronary heart disease + 0.358 × Diabetes + 0.511 × Pulmonary infection + 0.849 × Ventricular arrhythmia + 0.665 × Hyperlipidemia + 0.514 × Hypoalbuminemia + 0.055 × LVDD - 0.131 × LVEF. The model demonstrated an AUC of 0.780 (0.741–0.819), with an accuracy of 91.3%, and a specificity of 91.4%, indicating good predictive performance. Further validation through decision curve analysis and clinical impact curves confirmed the model’s effectiveness in clinical decision support.

**Conclusion:**

The study successfully developed a multivariate analysis-based prediction model capable of effectively predicting the risk of AMI in hospitalized elderly AHF patients. This model provides a powerful tool for clinicians, facilitating early identification and intervention in high-risk patients.

## Introduction

AHF is a life-threatening cardiovascular syndrome that typically occurs on the basis of pre-existing structural or functional cardiac abnormalities or chronic diseases (1). It is characterized by a sudden onset and rapid progression, often requiring urgent hospitalization. In recent years, the incidence of AHF has been steadily increasing, driven by an aging population and the rising prevalence of chronic conditions such as hypertension, diabetes mellitus, and coronary artery disease (CAD) (2). Patients with AHF often face a substantial risk of short-term mortality and a high rate of hospital readmissions, imposing a significant burden on patients, their families, and the healthcare system (3, 4). AMI is a serious cardiovascular event caused by the acute partial or complete occlusion of a coronary artery, leading to a sudden reduction in myocardial perfusion (5). Without prompt intervention, AMI can result in irreversible myocardial damage or even death. In individuals with AHF, whose hearts are already under significant hemodynamic stress and functional compromise, the likelihood of developing AMI is markedly increased. The occurrence of AMI in this context exacerbates myocardial ischemia, further impairs cardiac function, and significantly elevates the risk of mortality (6).

Cardiac biomarkers such as troponin I remain a critical and reliable tool for diagnosing AMI. However, in clinical practice, physicians managing elderly patients with AHF often focus primarily on treating heart failure symptoms—such as dyspnea and edema—while potentially overlooking signs of concurrent AMI. This challenge is compounded by the fact that older patients frequently lack classic ischemic symptoms like chest pain due to diminished pain perception (7). As a result, AMI may be missed or diagnosed late, leading to worse outcomes. In this context, a predictive model based on accessible clinical parameters can serve as an early warning tool to support clinicians in identifying high-risk patients even when typical signs are absent.

Given these diagnostic challenges in elderly AHF patients, it is concerning that research into effective AMI prediction tools remains scarce. Existing predictive models are often constrained by small sample sizes or inadequate variable selection, making their application in real-world clinical settings challenging. Moreover, many models fail to account for the atypical presentations of AMI in elderly AHF patients, resulting in under-recognition and treatment delays. In recent years, nomograms—a graphical representation of a predictive model—have emerged as practical tools in clinical decision-making due to their simplicity, visual clarity, and individualized risk estimation. By integrating multiple risk factors into a user-friendly format, nomograms allow physicians to rapidly assess patient-specific risk at the bedside, thereby improving diagnostic efficiency and personalized care. In this context, our study aims to incorporate key risk factors present at admission in AHF patients and construct a logistic regression–based nomogram to evaluate and quantify the risk of in-hospital AMI. This model is designed to facilitate early identification of high-risk individuals and support timely clinical decision-making. Rather than replacing established diagnostic markers such as cardiac troponin, it serves as a complementary tool—particularly useful when typical symptoms are absent or clinical suspicion is low. By stratifying AMI risk upon admission, the nomogram may prompt closer surveillance in high-risk patients, such as trending troponins, performing serial ECGs, or involving cardiology consultation earlier, thereby improving the likelihood of timely diagnosis and intervention.

## Materials and methods

### Study design and patients

This retrospective study included patients hospitalized in the Cardiovascular Department of Qinhuangdao City First Hospital from October 2019 to December 2023. Inclusion criteria were: (1) aged 65 years and older; (2) diagnosed with AHF according to the European Society of Cardiology guidelines; (3) patients with complete medical records, laboratory results, and other necessary medical documentation. Exclusion criteria included: (1) patients with AMI present at the time of admission, including those in whom AHF was secondary to AMI; (2) patients lacking complete medical records, laboratory results, or other essential clinical data; (3) patients not meeting the diagnostic criteria for AHF. AMI events included in this study were those newly developed during the hospitalization for AHF, as confirmed by medical records. Patients with AMI diagnosed at or prior to admission were excluded. Only events occurring after admission and before discharge were considered in-hospital AMI, as documented in the medical records.

### Ethical statement

This study was based on a retrospective analysis of existing case data. All patient data collection and analysis were conducted anonymously to ensure privacy protection. Additionally, the study was approved and supported by the Institutional Review Board of Qinhuangdao City First Hospital (Approval Number: 202401A111).

### Disease definitions

AHF is an urgent cardiac condition primarily characterized by a sudden decrease in the heart’s pumping function, rendering the circulatory system unable to supply sufficient blood to meet the metabolic demands of the body’s tissues and organs. The European Society of Cardiology defines it as a pathological state due to acute changes in cardiac structure or function resulting in elevated ventricular filling pressures and/or significant reductions in ejection fraction (2). Key clinical presentations include rapidly increased dyspnea, fatigue, and edema of the lower limbs. One of the critical diagnostic markers for AHF is the determination of serum levels of B-type natriuretic peptide (BNP) or its precursor, N-terminal pro-B-type natriuretic peptide (NT-proBNP). Diagnostic thresholds for AHF use different BNP and NT-proBNP levels adapted for various age groups. Specifically, the diagnostic threshold for BNP is ≥300 pg./mL for all age groups, while NT-proBNP thresholds are stratified by age: >450 pg./mL for patients under 55 years, >900 pg./mL for those aged 55 to 75, and >1800 pg./mL for those over 75 years (8).

AMI is an acute myocardial ischemic event typically caused by sudden obstruction of coronary artery blood flow, leading to an imbalance between myocardial supply and demand. According to the latest guidelines from the European Society of Cardiology (ESC) and the American Heart Association (AHA)/American College of Cardiology (ACC), the diagnosis of AMI relies on clinical symptoms (such as typical chest pain or discomfort possibly accompanied by nausea, vomiting, sweating, or dyspnea), dynamic changes in the electrocardiogram (ECG) (including ST-segment elevation or new onset of left bundle branch block, and possible ST-segment depression or T-wave inversion), elevated cardiac injury markers (such as Troponins T and I, or Creatine Kinase-MB levels more than twice the upper limit), and imaging evidence of new myocardial injury or dysfunction (9, 10).

### Data collection

This retrospective study collected data from patients hospitalized in the Cardiovascular Department of Qinhuangdao City First Hospital from October 2019 to December 2023. Data was gathered through the medical record system and included patient demographics such as gender, age, body mass index (BMI), smoking history, and prior medical history, which predominantly featured CAD, hypertension, prior stroke, chronic renal failure, diabetes, chronic obstructive pulmonary disease (COPD), cancer, atrial fibrillation, and heart valve disease. Complications during hospitalization primarily included pulmonary infections, ventricular arrhythmias, acute kidney injury, stress ulcers, with tests and examinations covering hemoglobin, electrolytes, duplex ultrasonography of the lower limbs, and echocardiography, among other relevant data.

### Model development

#### Dataset configuration and variable selection

In this study, we employed logistic regression to predict the occurrence of AMI during hospitalization in elderly patients with AHF. Initially, the Least Absolute Shrinkage and Selection Operator (LASSO) method was used to screen the collected data, identifying significant risk factors related to AMI. Subsequently, these factors were evaluated using a multivariate logistic regression model to ensure that only statistically significant predictors were included in the final model. A nomogram was constructed to make the model’s predictions and the contributions of each variable transparent and easy to understand.

#### Model evaluation and interpretation

During the evaluation phase, the model’s discriminative ability was assessed by calculating the Area Under the Curve (AUC), while calibration was evaluated using a bootstrap-based calibration curve (1,000 bootstrap resamples), which provides a more reliable assessment of model fit, especially for large sample sizes. The discrimination ability of the model was assessed using the concordance statistic (C-statistic). The C-statistic quantifies the probability that, in a randomly selected pair of patients—one who experienced the outcome and one who did not—the model assigns a higher predicted risk to the patient who experienced the event. A C-statistic of 0.5 indicates no discriminative power (equivalent to chance), whereas a value of 1.0 indicates perfect discrimination. Generally, a C-statistic above 0.7 is considered acceptable model performance. Additionally, Decision Curve Analysis (DCA) was applied to calculate the net benefit across different thresholds to comprehensively evaluate the model’s practical value in clinical decision-making. Clinical Impact Curves (CIC) were introduced to visualize the impact of the model’s predictions on actual clinical interventions, such as treatments at different threshold settings, optimizing the model’s clinical application while ensuring maximum health benefits with minimal unnecessary medical interventions. This strategy aims to build an accurate and highly interpretable model, providing a robust tool for clinicians to identify the risk of AHF in elderly patients earlier.

### Statistical analysis

All statistical analyses in this study were performed using SPSS version 24.0 and the R language. The significance level was set at *p* < 0.05. Descriptive statistics analyzed the baseline information of the participants. The normality of continuous variables was verified using the Kolmogorov–Smirnov test. Variables conforming to a normal distribution were described as mean ± standard deviation, while those not normally distributed were described using the median and interquartile range. Categorical variables were presented in frequencies and percentages. Variance Inflation Factor (VIF) and tolerance were calculated to assess potential collinearity among the parameters, with a VIF value below 5 and tolerance above 0.1 considered indicative of no significant collinearity.

## Results

### Baseline characteristics of patients

**Figure 1:**
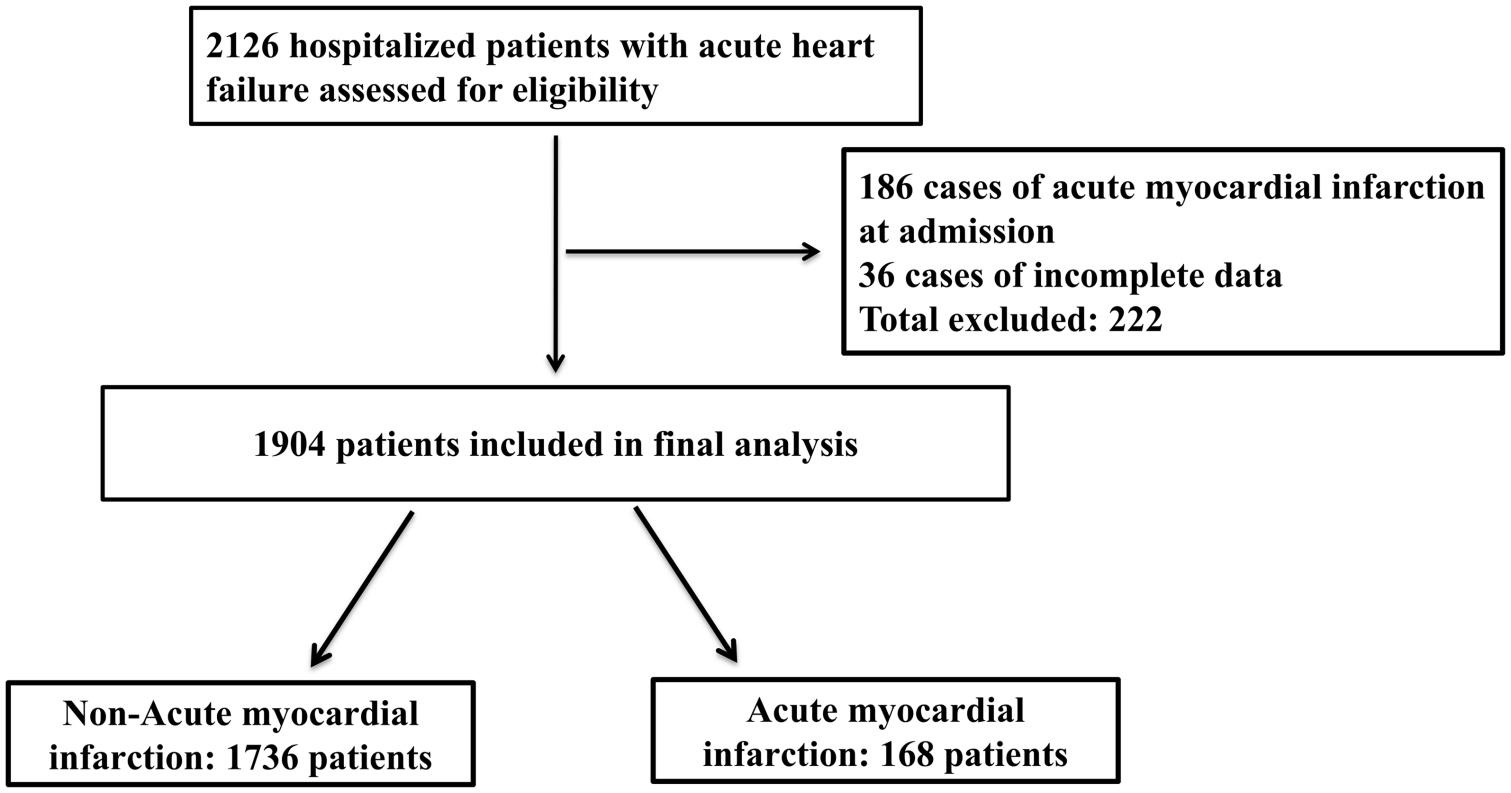
The patient flow chart in our study.

Between October 2019 and December 2023, we included a total of 2,126 elderly patients with AHF. After excluding 186 patients with AMI at admission and 36 patients with incomplete data (a total of 222 exclusions), 1,904 patients were included in the final cohort (Figure 1). Table 1 presents the baseline clinical characteristics of the elderly AHF patients, differentiated into AMI and non-AMI groups. Overall, the average age was 73.72 ± 6.01 years, with 1,088 males (57.1%) and 816 females (42.9%). There were 168 cases (8.82%) of AMI occurring during hospitalization. Significant statistical differences in age were observed between the two groups. The prevalence of CAD and diabetes was significantly higher in the AMI group compared to the non-AMI group (*p* < 0.05). Additionally, complications such as pulmonary infections and ventricular arrhythmias also showed a higher incidence in the AMI group, with statistically significant differences (*p* < 0.05).

**Table 1:** Baseline clinical characteristics of AHF patients classified by AMI.

Variables	Total (*N* = 1904)	Non-AMI (*N* = 1736)	AMI (*N* = 168)	*p*-value
Gender, *N* (%)				
Male	1,088(57.1%)	989(57.0%)	99(58.9%)	0.624
Female	816(42.9%)	747(43.0%)	69(41.1%)	
Age, mean ± SD (years)	73.72 ± 6.01	73.5 ± 5.84	75.99 ± 7.19	<0.001
BMI (kg/m^2^)	23.55 ± 3.12	23.90 ± 3.34	23.52 ± 3.10	0.124
Smoking				0.835
Yes	621(32.6%)	565(32.5%)	56(33.3%)	
No	1,283(66.7%)	1,171(67.5%)	112(66.7%)	
Comorbidity *N* (%)				
Coronary heart disease				0.003
Yes	643(33.8%)	569(32.8%)	74(44.0%)	
No	1,261(66.2%)	1,167(67.2%)	94(56.0%)	
Hypertension				0.923
Yes	617(32.4%)	562(32.4%)	55(32.7%)	
No	1,287(67.6%)	1,174(67.6%)	113(67.3%)	
Old cerebral infarction				0.609
Yes	646(33.9%)	586(33.8%)	60(35.7%)	
No	1,258(66.1%)	1,150(66.2%)	108(64.3%)	
Chronic renal failure				0.958
Yes	213(11.2%)	194(11.2%)	19(11.3%)	
No	1,691(88.8%)	1,542(88.8%)	149(88.7%)	
Diabetes				0.018
Yes	562(29.5%)	499(28.7%)	63(37.5%)	
No	1,342(70.5%)	1,237(71.3%)	105(62.5%)	
COPD				0.803
Yes	260(13.7%)	236(13.6%)	24(14.3%)	
No	1,644(86.3%)	1,500(86.4%)	144(85.7%)	
Cancer				0.693
Yes	69(3.6%)	62(3.6%)	7(4.2%)	
No	1835(96.4%)	1,674(96.4%)	161(95.8%)	
Heart valve disease				0.389
Yes	546(28.7%)	493(28.4%)	53(31.5%)	
No	1,358(71.3%)	1,243(71.6%)	115(68.5%)	
Atrial fibrillation				0.972
Yes	259(13.6%)	236(13.6%)	23(13.7%)	
No	1,645(86.4%)	1,500(86.4%)	145(86.3%)	
Complications				
Pulmonary infection				0.002
Yes	297(15.6%)	256(14.7%)	41(24.4%)	
No	1,607(84.4%)	1,480(85.3%)	127(75.6%)	
Ventricular arrhythmia				<0.001
Yes	242(12.7%)	201(11.6%)	41(24.4%)	
No	1,662(87.3%)	1,535(88.4%)	127(75.6%)	
Acute kidney Injury				0.59
Yes	433(22.7%)	392(22.6%)	41(24.4%)	
No	1,471(77.3%)	1,344(77.4%)	127(75.6%)	
Stress ulcer				0.975
Yes	11(0.6%)	10(0.6%)	1(0.6%)	
No	1893(99.4%)	1726(99.4%)	167(99.4%)	

Values are presented as mean ± standard deviation, median (interquartile range), or number (percentage) as appropriate, SD Standard deviation, BMI Body Mass Index, COPD Chronic Obstructive Pulmonary Disease.

### Univariate analysis of laboratory data and ultrasound examinations

Table 2 displays the laboratory tests, cardiac ultrasound, and lower limb venous ultrasound characteristics of elderly AHF patients. The incidence of hyperlipidemia and hypoalbuminemia was significantly higher in the AMI group compared to the non-AMI group, showing a significant statistical difference (*p* < 0.05). Additionally, the ejection fraction was notably lower and the LVDD was significantly larger in the AMI group than in the non-AMI group, with these differences also being statistically significant (*p* < 0.05).

**Table 2:** The results of univariate analysis of laboratory data and ultrasound examination.

Variables	Total (*N* = 1904)	Non-AMI (*N* = 1736)	AMI (*N* = 168)	*p*-value
Hypertriglyceridemia				0.292
Yes	340(17.9%)	305(17.6%)	35(20.8%)	
No	1,564(82.1%)	1,431(82.4%)	133(79.2%)	
Hyperlipemia				<0.001
Yes	989(51.9%)	876(50.5%)	113(67.3%)	
No	915(48.1%)	860(49.5%)	55(32.7%)	
Anemia				0.454
Yes	216(11.3%)	194(11.2%)	22(13.1%)	
No	1,688(88.7%)	1,542(88.8%)	146(86.9%)	
Hypokalemia				0.044
Yes	426(22.4%)	378(21.8%)	48(28.6%)	
No	1,478(77.6%)	1,358(78.2%)	120(71.4%)	
Hyponatremia				0.478
Yes	239(12.6%)	215(12.4%)	24(14.3%)	
No	1,665(87.4%)	1,521(87.6%)	144(85.7%)	
Hypoalbuminemia				0.017
Yes	417(21.9%)	368(21.2%)	49(29.2%)	
No	1,487(78.1%)	1,368(78.8%)	119(70.8%)	
LVDD	57.25 ± 4.72	57.15 ± 4.64	58.33 ± 5.43	0.002
LVEF	41.49 ± 6.29	41.93 ± 6.08	36.96 ± 6.72	<0.001
Lower extremity venous thrombosis				0.804
Yes	215(11.3%)	197(11.3%)	18(10.7%)	
No	1,689(88.7%)	1,539(88.7%)	150(89.3%)	

Values are presented as median (interquartile range), or number (percentage) as appropriate, LVDD left ventricular end diastolic dimension, LVEF left ventricle ejection fraction.

### Development and validation of the nomogram

**Figure 2:**
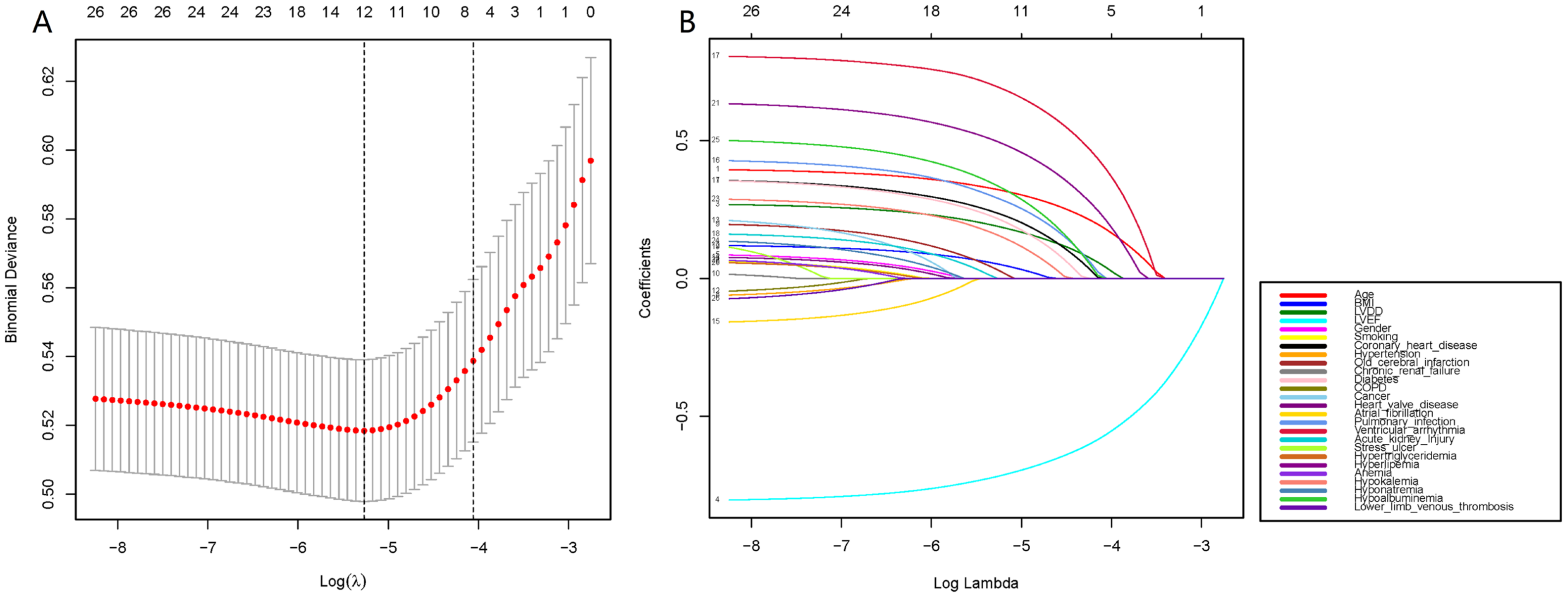
Data statistics and clinical feature selection using the LASSO binary logistic regression model. **(A)** LASSO coefficient profiles of the 26 features. A coefficient profile plot was produced against the log(lambda) sequence. **(B)** Optimal parameter (lambda) selection in the LASSO model used fivefold cross-validation via minimum criteria. The partial likelihood deviance (binomial deviance) curve was plotted versus log(lambda).

**Figure 3:**
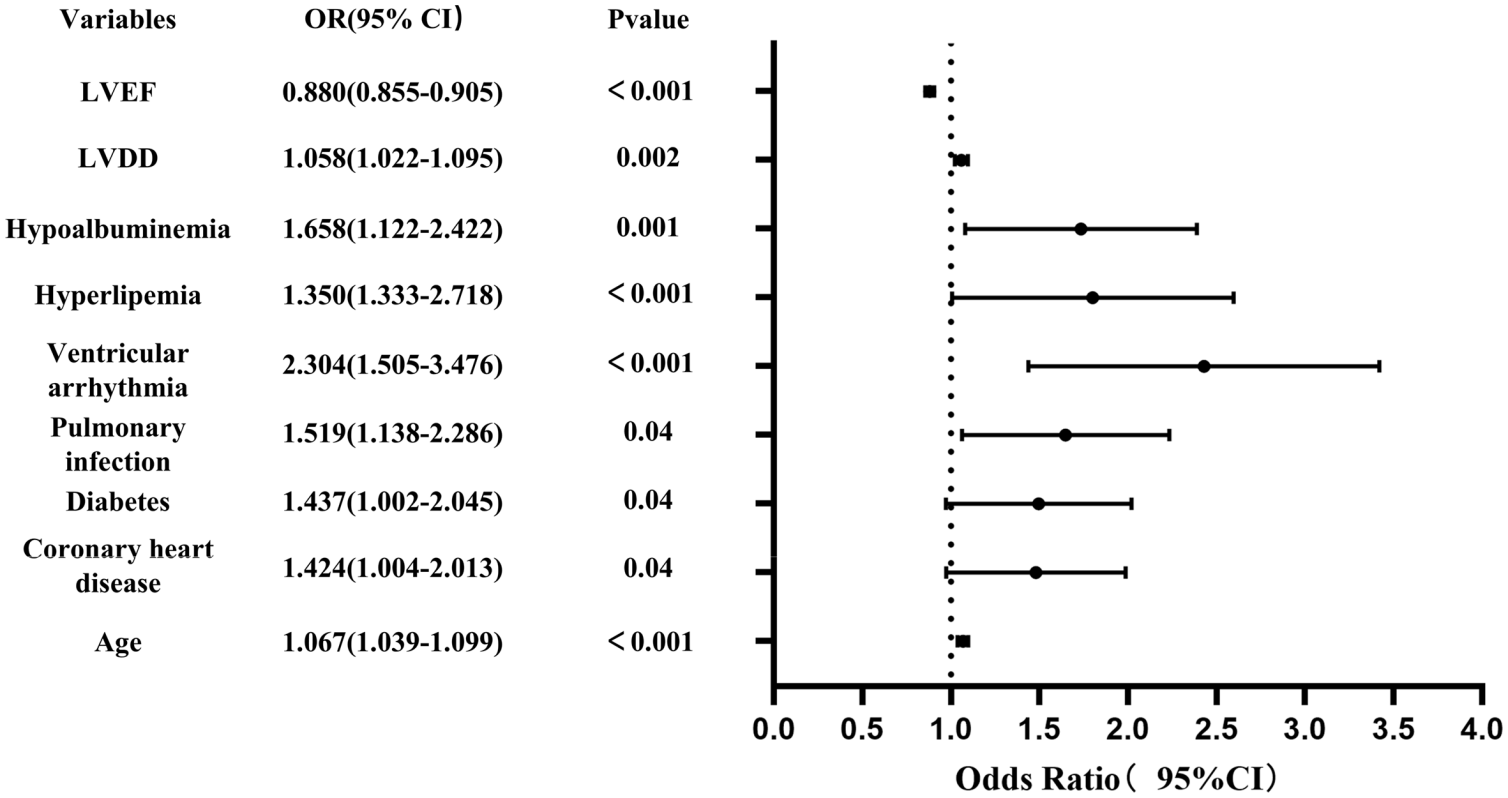
Forest plot showing the relationship between risk factors and the occurrence of AMI in elderly with acute heart failure.

**Figure 4:**
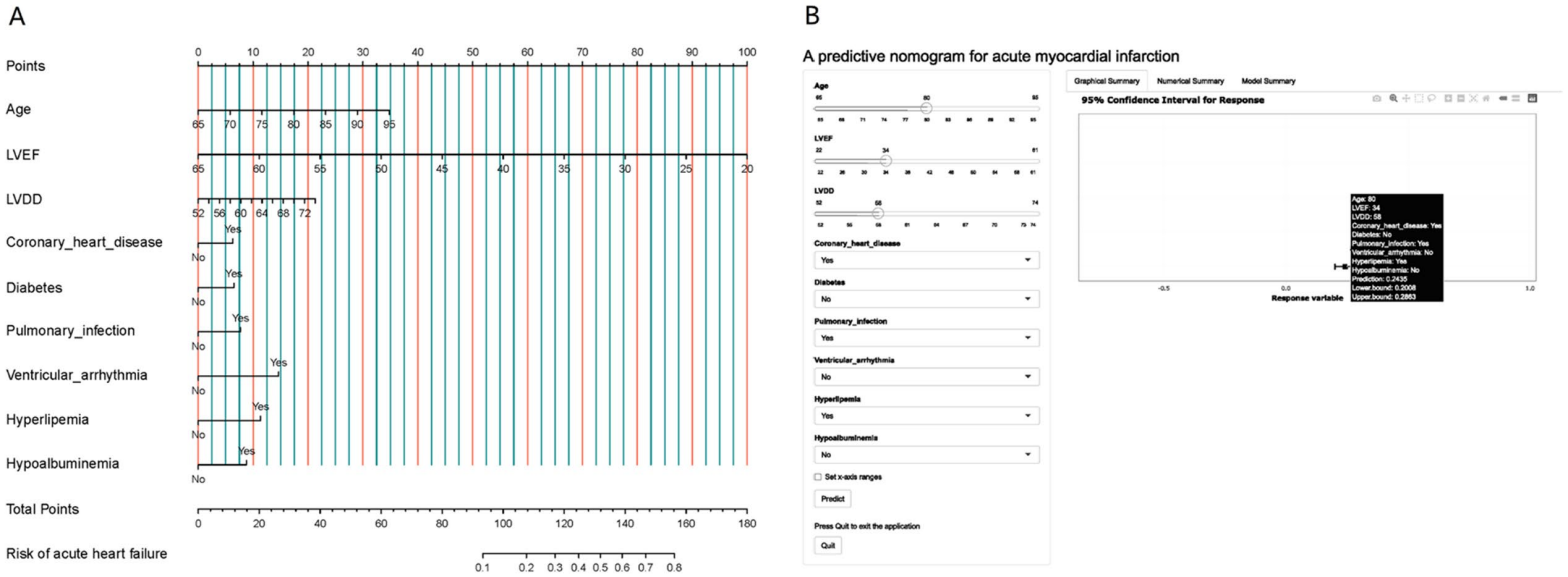
**(A)** A nomogram designed to calculate the risk of AMI in elderly patients hospitalized with AHF. This tool incorporates variables such as age, LVEF, LVDD, presence of coronary heart disease, diabetes, pulmonary infection, ventricular arrhythmia, hyperlipemia, and hypoalbuminemia. The nomogram assigns points for each parameter based on its contribution to the risk of AMI, allowing clinicians to add up these points to estimate a patient’s risk level. **(B)** Presented is an online dynamic version of the nomogram, hosted at https://longmao.shinyapps.io/longmao1/, which offers an interactive approach to assessing the probability of AMI during hospitalization for elderly AHF patients. It features an example case with a filled set of parameters, including an 80-year-old patient with a LVEF of 34, LVDD of 68, and conditions such as coronary heart disease, pulmonary infection, and hyperlipemia. The online tool calculates the risk of AMI and provides a prediction with a 95% confidence interval.

**Figure 5:**
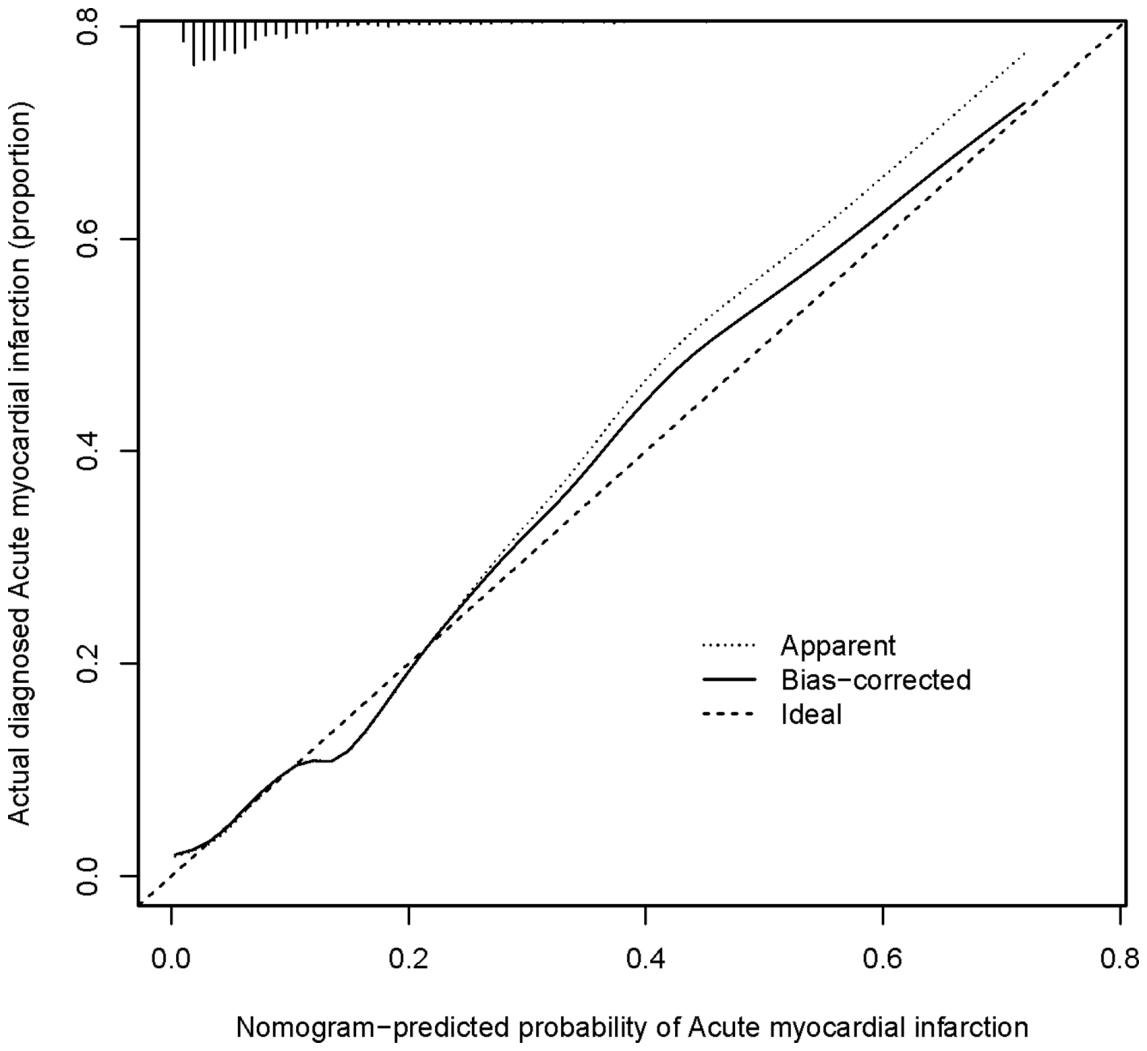
A calibration curve for a nomogram used to predict the risk of acute heart failure in a cohort The *x*-axis shows the predicted risk of acute heart failure as estimated by the nomogram. The *y*-axis indicates the actual diagnosed incidence of acute heart failure. The diagonal dotted line on the graph shows what a perfect prediction would look like, where the predicted risk of acute heart failure matches exactly with what actually happened. The solid line shows how well the nomogram is predicting. If the solid line is very close to the dotted line, it means the nomogram’s predictions are accurate.

Using the R language, we first performed LASSO regression on the training set of the included population, selecting 12 variables out of 26 (Figures 2A,B). The selected variables were age, BMI, LVDD, LVEF, coronary heart disease, old cerebral infarction, diabetes, pulmonary infection, ventricular arrhythmia, hyperlipidemia, hypokalemia, and hypoalbuminemia. Multifactorial logistic regression analysis identified age, coronary heart disease, diabetes, pulmonary infection, ventricular arrhythmia, hyperlipidemia, hypoalbuminemia, LVDD, and LVEF as independent risk factors for the occurrence of AMI in elderly AHF patients during hospitalization (Table 3 and Figure 3). Based on these independent risk factors, we constructed a nomogram model diagram to predict the probability of AMI occurrence during hospitalization in elderly AHF patients (Figure 4). This predictive tool is also available for online inquiry and utilization at https://longmao.shinyapps.io/longmao1/ (Figure 4B). The predictive model is expressed as: Logit(P) = −7.286 + 0.065 × Age + 0.380 × Coronary heart disease + 0.358 × Diabetes + 0.511 × Pulmonary infection + 0.849 × Ventricular arrhythmia + 0.665 × Hyperlipidemia + 0.514 × Hypoalbuminemia + 0.055 × LVDD − 0.131 × LVEF. The variance inflation factors (VIF) for each variable in the model were calculated, indicating that all predictor VIF values were well below the threshold of 5, suggesting no significant collinearity (Age 1.01, Coronary heart disease 1.01, Diabetes 1.00, Pulmonary infection 1.00, Ventricular arrhythmia 1.01, Hyperlipidemia 1.00, Hypoalbuminemia 1.02, LVEF 1.01, LVDD 1.02). Using the bootstrap method, 1,000 resamples were performed to assess the nomogram. The results showed that the calibration curve of the nomogram had only minor deviations compared to the ideal prediction line, demonstrating high consistency between the model’s predictions and the observed data (Figure 5).

**Table 3:** Prediction factors of AMI in geriatric patients with AHF.

	*B*	Odds ratio	*p* value	95%CI
Age	0.065	1.067	<0.001	1.039–1.099
Coronary heart disease	0.354	1.424	0.04	1.004–2.013
Diabetes	0.362	1.437	0.04	1.002–2.045
Pulmonary infection	0.418	1.519	0.04	1.138–2.286
Ventricular arrhythmia	0.835	2.304	<0.001	1.505–3.476
Hyperlipemia	0.639	1.350	<0.001	1.333–2.718
Hypoalbuminemia	0.300	1.658	0.001	1.122–2.422
LVDD	0.056	1.058	0.002	1.022–1.095
LVEF	−0.128	0.880	<0.001	0.855–0.905
Constant	−7.464	0.001	<0.001	

LVDD, left ventricular end diastolic dimension; LVEF, left ventricle ejection fraction; B, regression coefficient; 95%CI, 95% confidence interval.

**Figure 6:**
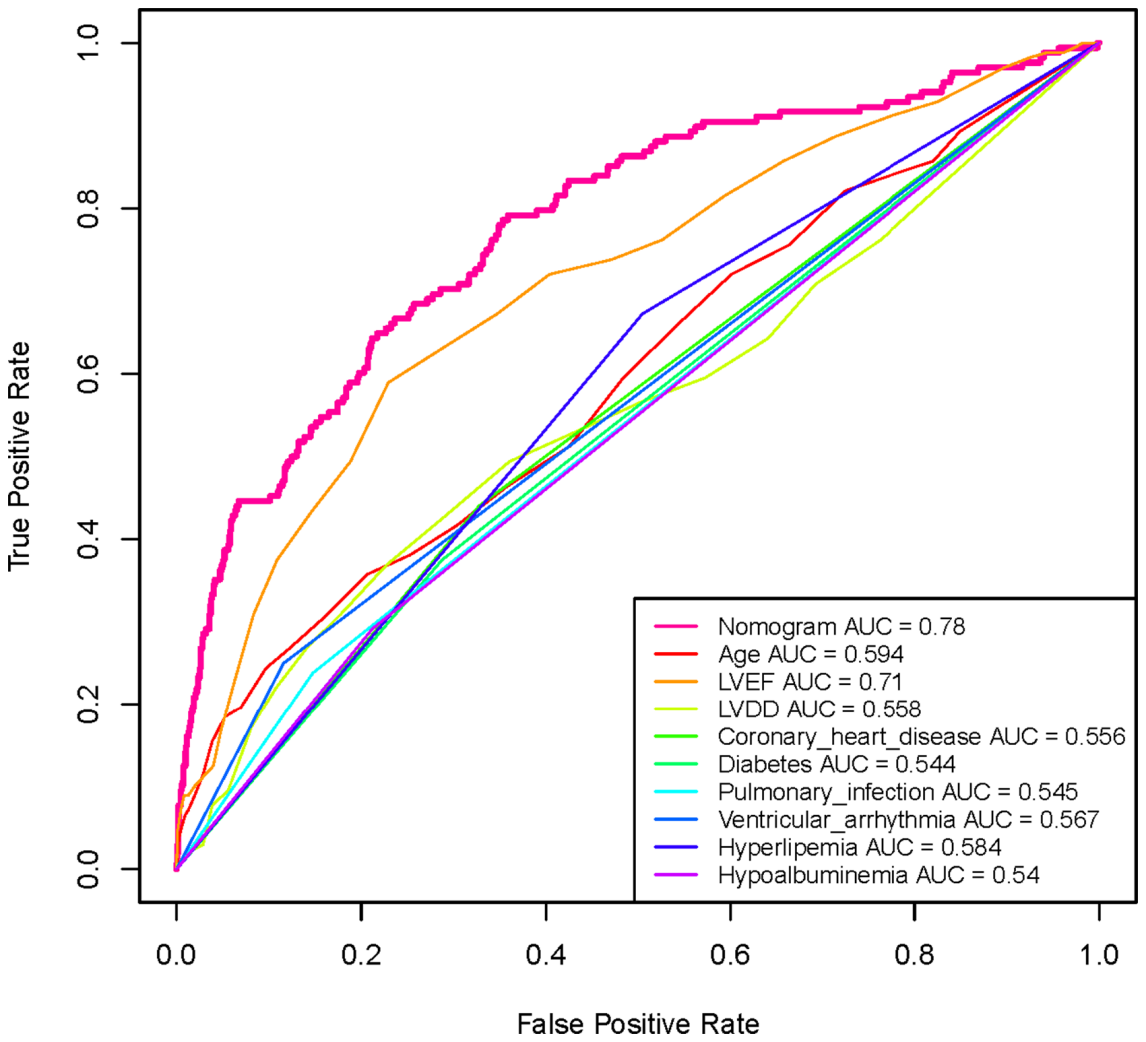
Analysis of the ROC curve for the predictive values of AMI in elderly patients with acute heart failure. The Nomogram curve is the most reliable predictor with the highest AUC value of 0.78, indicating its superior performance in forecasting the occurrence of AMI among the given factors. This curve significantly outperforms the other individual factors like age, LVEF, LVDD, coronary heart disease, diabetes, and others listed in the legend, whose AUC values range from 0.544 to 0.71. The higher AUC of the Nomogram suggests a greater accuracy in distinguishing between patients who are likely and unlikely to suffer from an AMI.

**Figure 7:**
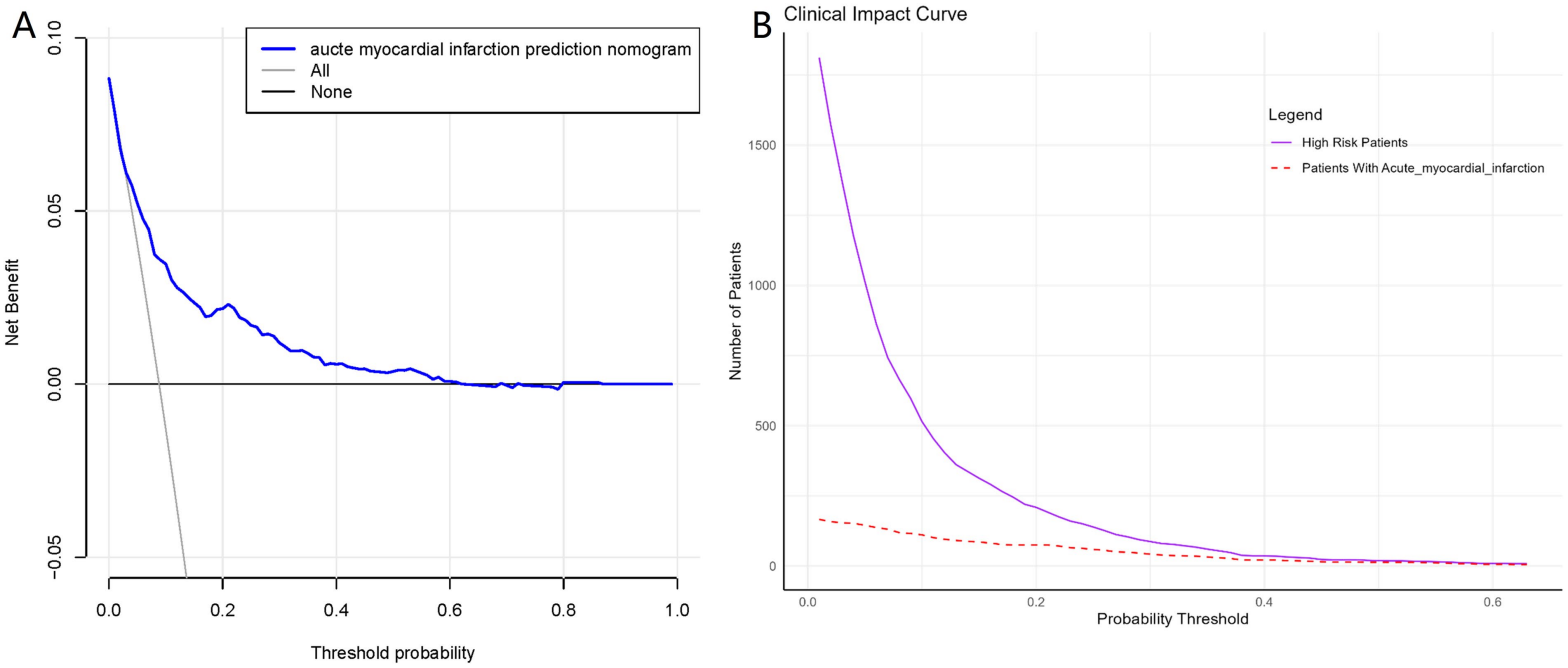
Decision curve analysis and Clinical Impact Curves for the myocardial infarction nomogram. **(A)** Shows a DCA for the AMI prediction nomogram. The blue line shows the benefit of using the nomogram at various thresholds, indicating when it’s useful. Part **(B)** is the CIC, which demonstrates the clinical applicability of the nomogram. The solid purple line counts patients identified as high risk by the nomogram, while the dashed red line shows how many actually have the condition.

The ROC curve was constructed, and the AUC for the nomogram was 0.780 (95% CI, 0.741–0.819), demonstrating significant superiority over other variables (Figure 6). Internal validation using 1,000 bootstrap resamples yielded a C-index of 0.769, indicating good discriminative performance of the model. This indicates that the model has a reasonable ability to distinguish patients at high risk of developing AMI during hospitalization for AHF, as demonstrated by its AUC and C-index values. DCA and CIC revealed the practical application of the predictive model (Figures 7A,B). DCA showed that within the probability threshold range of 1 to 63%, the model provides meaningful clinical decision support. Specifically, within this range, the net benefit is significant, indicating a higher likelihood of patients benefiting from treatment interventions suggested by the model and a lower risk of unnecessary treatment interventions. The CIC illustrated how the predictive model impacts the number of patients expected to receive treatment across different probability thresholds. The solid line represents the number of all high-risk patients, while the dashed line shows the number of patients who indeed suffered an AMI as predicted by the model. Clearly, the model identifies most patients likely to suffer a myocardial infarction at lower thresholds. Risk assessment through DCA and CIC assists in developing personalized treatment plans for elderly patients with AMI. Using the model’s predictions, physicians can achieve a better balance in treatment decisions, maximizing the reduction of adverse cardiovascular events while avoiding excessive interventions in low-risk patients.

Table 4 displayed the model’s accuracy, sensitivity, and specificity, demonstrating its superior predictive performance, especially in terms of accuracy and specificity. The model’s accuracy reached 91.3%, indicating its capability to accurately diagnose whether a patient will suffer an AMI. Notably, the specificity was as high as 91.4%, ensuring high reliability in identifying patients who will not suffer a myocardial infarction, with almost no false positives. Although the sensitivity was relatively lower at 62.5%, this result is still within an acceptable range and could be improved with further model tuning. However, it should be noted that the relatively low sensitivity (62.5%) suggests that the model has limitations in detecting all true-positive AMI cases. This means that while the model performs well in terms of accuracy and specificity, it may still miss some high-risk patients.

**Table 4:** Predictive performance of the logistic regression model for in-hospital AMI in Elderly Patients with AHF.

	AUC	Accuracy	Sensitivity	Specificity
LR	0.780	91.3%	62.5%	91.4%

LR, logistic regression.

## Discussion

In this study, we retrospectively analyzed the clinical data of hospitalized patients with AHF to develop a predictive model for assessing the risk of in-hospital AMI. A total of 1,904 eligible patients were included. Univariate and multivariate logistic regression analyses were conducted to identify independent risk predictors, which included age, history of CAD, diabetes mellitus, pulmonary infection, ventricular arrhythmia, hyperlipidemia, hypoalbuminemia, LVDD, and LVEF. Based on these variables, we constructed a multivariate logistic regression model and developed a corresponding nomogram to visually illustrate the contribution of each risk factor to AMI risk. To enhance the model’s accessibility and usability in clinical practice, we further developed an interactive web-based application.^1^ This tool allows clinicians to input patient-specific data online and instantly obtain individualized AMI risk estimates. The application not only improves the practical utility of the model but also provides healthcare professionals with a user-friendly, intuitive platform for risk assessment and clinical decision support. Internal validation demonstrated that the model had good discriminative performance (AUC = 0.780) and calibration accuracy, indicating a high level of predictive reliability. DCA further confirmed its clinical applicability. However, existing clinical assessment methods for predicting in-hospital AMI in AHF patients largely rely on the presence of overt clinical symptoms and standard biomarker or ECG changes. Our model addresses this clinical gap by enabling proactive risk stratification before the onset of typical manifestations. The clinical significance of this model lies in its ability to identify high-risk patients before they present with overt symptoms of AMI. Given the significantly elevated mortality risk once AMI occurs in AHF patients, early risk stratification is essential. By utilizing the nomogram and web-based tool, clinicians can prioritize monitoring and management for these high-risk individuals, such as increased frequency of cardiac biomarker testing, dynamic ECG monitoring, and repeated echocardiographic assessment in patients flagged as high risk. Early identification of subtle signs of AMI allows for prompt intervention, which may not only improve immediate treatment outcomes and reduce the incidence of in-hospital adverse cardiovascular events, but also support more informed decisions regarding hospitalization duration and the intensity of clinical surveillance, ultimately contributing to better long-term prognosis and functional recovery.

One limitation of this study is the imbalanced nature of the dataset, with AMI patients constituting only 8.8% of the total cohort, while the majority of patients are non-AMI (91.2%). This imbalance likely contributed to the model’s lower sensitivity of 62.5%, as the model may have been more inclined to predict the non-AMI outcomes, given their higher prevalence. This can reduce the model’s ability to effectively identify high-risk AMI patients. Although this imbalance was not addressed through resampling techniques such as SMOTE or weighted loss functions in the current study, we acknowledge that these methods could potentially improve the model’s sensitivity. However, for the current analysis, we chose to keep the original distribution of the dataset without such interventions. In future studies, we plan to explore the use of data resampling methods (e.g., SMOTE) or weighted loss functions to better handle data imbalance, which may enhance the model’s sensitivity and improve its clinical applicability. Additionally, adjusting for imbalanced data could further refine the model’s performance, especially in identifying high-risk AMI patients.

Age is a significant risk factor for AMI, especially in the elderly population. Atherosclerosis intensifies in older individuals, and as age progresses, the severity of atherosclerosis increases, leading to gradual narrowing and hardening of the coronary arteries. This narrowing restricts blood flow, increasing the risk of myocardial infarction. The accumulation of lipids and calcium within the arterial walls is a direct cause of the high incidence of AMI in the elderly (11). With aging, the prevalence of complications from cardiovascular diseases such as hypertension, diabetes, and hyperlipidemia increases, each of which is an independent risk factor for AMI. Common chronic conditions in the elderly negatively affect overall heart function, further increasing the risk of AMI (12, 13). The elderly have a diminished response to acute physiological stress, including vascular reactivity and heart rate regulation. This weakened physiological mechanism means that during acute coronary events, the elderly have insufficient physiological adaptability to effectively manage drastic changes in blood flow. Studies have shown that elderly individuals have higher levels of inflammation, and chronic inflammation is a key driver of atherosclerosis and cardiovascular disease (14). Thus, sustained low-level systemic inflammation may accelerate the progression of CAD and increase the risk of AMI. Therefore, clinicians need to be highly vigilant when treating elderly patients, and developing comprehensive management strategies is key to improving treatment outcomes and preventing the occurrence of AMI.

CAD is recognized as a major risk factor for AMI. The hallmark of CAD is the accumulation of plaques within the coronary artery walls, composed of lipids, cholesterol, and other cells. Over time, these plaques can thicken and harden, leading to the narrowing of the vessels. This narrowing restricts blood flow, decreases myocardial oxygen supply, and increases the risk of myocardial infarction (15, 16). Patients with AHF often experience pulmonary congestion and tissue hypoxia, which can trigger an inflammatory response. Additionally, heart failure can lead to endothelial dysfunction and abnormalities in immune function, further promoting inflammation. Research indicates that inflammation not only promotes plaque formation but can also accelerate plaque instability, making them more likely to rupture. The ruptured plaque surface can initiate platelet aggregation and thrombus formation (17, 18). This process may result in rapid, complete blockage of the coronary artery, triggering an AMI. Therefore, for patients with a definitive diagnosis of CAD who develop AHF, doctors need to take proactive preventive measures, including medication and lifestyle adjustments, to manage and reduce the occurrence of AMI.

In patients with AHF who also have diabetes, the risk of AMI significantly increases. Diabetes itself enhances the risk of cardiovascular events by accelerating atherosclerosis, enhancing platelet aggregation, and impairing endothelial function (19). When this metabolic disorder coexists with AHF, the heart’s stress capacity is further diminished, leading to insufficient blood supply under heavy cardiac workload conditions, thus triggering AMI (20). The hyperglycemic state in diabetic patients not only damages the vascular endothelium but also leads to systemic inflammation and vascular dysfunction, all of which are significant factors in the worsening of heart failure (21). Moreover, the metabolic abnormalities caused by diabetes can exacerbate myocardial energy metabolism insufficiency, increasing the likelihood of myocardial hypoxia. Poor glycemic control in diabetic patients intensifies cardiac metabolic imbalance. In the context of heart failure, the heart’s demand for oxygen and energy increases, while hyperglycemia and insulin resistance induced by diabetes restrict the heart’s ability to adapt to these needs, making the heart more sensitive to acute coronary events, increasing the possibility of myocardial ischemia and myocardial cell damage (22, 23). For patients with AHF complicated by diabetes, it is crucial clinically to closely monitor the risk of myocardial infarction and take preventive measures such as optimizing glycemic control and strictly managing other cardiovascular risk factors to reduce the occurrence of AMI (24). This comprehensive management strategy is vital for improving the prognosis of these high-risk patients.

During hospitalization, elderly patients with AHF who also contract a pulmonary infection experience a significant imbalance between oxygen demand and supply, as pulmonary infections can decrease gas exchange efficiency, causing hypoxemia and increasing the heart’s oxygen demand (25). In the context of heart failure, where there is already an insufficient oxygen supply to the heart, a pulmonary infection can exacerbate this condition and increase the risk of myocardial infarction. The systemic inflammatory response caused by pulmonary infections can exacerbate cardiovascular system stress through various mechanisms (26). Elevated inflammatory factors such as cytokines can promote thrombus formation and accelerate the progression of CAD, thereby triggering AMI (27). Patients with lung infections often present with respiratory distress and hypoxemia, which forces the heart to increase its pumping action to meet the body’s oxygen demands. This can lead to excessive cardiac workload in patients with heart failure, increasing the risk of myocardial ischemia and infarction (28, 29). Therefore, in patients with AHF, the occurrence of myocardial infarction should be vigilantly monitored when a pulmonary infection is present. Clinically, these patients require close monitoring and management, optimization of oxygen therapy, infection control, and timely adjustment of cardiovascular medications to reduce the risk of myocardial infarction. Such a comprehensive treatment strategy is crucial for improving patient outcomes.

Patients with AHF often experience ventricular arrhythmias, significantly increasing their risk of AMI. Ventricular arrhythmias, such as ventricular tachycardia or fibrillation, reflect cardiac electrophysiological instability, which may be caused by myocardial ischemia, electrolyte imbalances, or myocardial damage (30). In the context of AHF, these abnormalities may indicate an impending myocardial infarction. AHF results in insufficient cardiac pumping function, reducing myocardial oxygen supply. Frequent or sustained ventricular arrhythmias can lead to a decrease in effective cardiac output, placing an additional burden on an already strained heart due to heart failure and exacerbating the supply–demand imbalance, thereby increasing the risk of AMI (31). Therefore, for patients with AHF and concomitant ventricular arrhythmias, clinical monitoring of cardiac status should be intensive, and any signs of potential myocardial ischemia should be promptly identified and managed. Medical interventions such as electrophysiological studies, antiarrhythmic medications, or electrical cardioversion should be employed to stabilize the rhythm and reduce the risk of AMI. Such heightened vigilance and proactive management are vital for improving survival rates in these high-risk patients.

Hyperlipidemia, especially high levels of LDL cholesterol and low levels of HDL cholesterol, accelerates atherosclerosis, leading to the narrowing or occlusion of coronary arteries, thereby increasing the risk of myocardial infarction (32). Hypoalbuminemia, particularly low levels of albumin, affects plasma colloid osmotic pressure, which may cause tissue edema and increase cardiac burden, and is associated with a chronic inflammatory state, affecting heart structure and function, thus increasing the risk of myocardial ischemia (33). In patients with AHF, hyperlipidemia and hypoalbuminemia can exacerbate the metabolic and functional burden on the heart, and can trigger a systemic inflammatory response, accelerating cardiac pathology through mechanisms such as thrombosis formation and vascular dysfunction, thereby increasing the risk of AMI. An increase in left ventricular end-diastolic diameter and a decrease in LVEF are often interrelated in patients with AHF. An increase in left ventricular end-diastolic diameter typically reflects elevated cardiac filling pressures and ventricular dilation, which may lead to increased myocardial tension and oxygen consumption, thus exacerbating myocardial ischemia (34). Meanwhile, a decrease in LVEF indicates a weakened cardiac pumping ability, reduced cardiac output, further exacerbating myocardial oxygen supply insufficiency. When these conditions coexist in AHF patients, the imbalance between the cardiac stress state and metabolic demands versus its oxygen supply capacity becomes more pronounced, greatly increasing the risk of AMI (35). Studies show that these changes in left ventricular structure and function in AHF patients are closely related to the progression of CAD, which may trigger or exacerbate myocardial infarction (36). Therefore, for patients with AHF, optimizing lipid levels and improving nutritional status is crucial, and clinicians should closely monitor cardiovascular status in patients with increased left ventricular end-diastolic diameter and decreased LVEF, and adjust treatment strategies timely to reduce the risk of AMI.

Despite the promising predictive performance of the model, the relatively low sensitivity of 62.5% suggests that the model may fail to detect all high-risk patients, which could lead to missed opportunities for early intervention. This limitation should be addressed before considering clinical deployment. Further optimization of the model, particularly to handle imbalanced data, will be necessary to enhance its sensitivity and overall clinical utility.

## Limitations

This study faces several key limitations that require cautious interpretation of its results. Firstly, this is a single-center retrospective study involving case data from only one hospital, not externally validated, which may affect the study’s general applicability and reliability. Secondly, due to its design as a non-random retrospective analysis, there is a risk of potential selection and inclusion biases. Although efforts were made to control these biases, they could not be entirely eliminated from affecting the study outcomes. Additionally, the study analysis is limited by the available data and may have omitted other key variables, which could affect the accuracy of the final conclusions. Lastly, the study focuses only on the risk of AMI during hospitalization in elderly patients with heart failure, not covering long-term prognosis and other relevant risk factor assessments. Future studies should enhance the predictive power of the research model by expanding the sample size, incorporating more variables, and conducting long-term follow-ups to overcome these limitations. Despite these limitations, the insights provided by this study are still of significant value.

## Conclusion

This study effectively predicts the risk of AMI during hospitalization in elderly patients with AHF through a logistic regression model. The model considers several clinically significant factors, including age, CAD, diabetes, and its high predictive accuracy provides important decision support for clinicians. Through extensive statistical validation, including AUC values and the Hosmer-Lemeshow test, the study demonstrates the model’s practical value and reliability in a clinical setting. The application of this risk assessment tool enables physicians to identify high-risk patients earlier, optimize treatment strategies, reduce unnecessary medical interventions, thereby significantly improving patient survival rates and quality of life, and advancing understanding of the mechanisms of AHF and innovation in future clinical practice.

## Data Availability

The raw data supporting the conclusions of this article will be made available by the authors, without undue reservation.

## Footnotes

^1^ https://longmao.shinyapps.io/longmao1/
