# Correction: Developing a predictive nomogram for AMI in elderly patients with AHF: a retrospective analysis

**DOI:** 10.3389/fmed.2025.1685604

**Published:** 2025-08-29

**Authors:** Frontiers Production Office

**Affiliations:** Frontiers Media SA, Lausanne, Switzerland

**Keywords:** acute myocardial infarction, acute heart failure, elderly, nomogram, prediction model

The ethics statement was erroneously given as “The studies involving humans were approved by the ethical review board of The First Hospital of Qinhuangdao, Qinhuangdao, Hebei, China, evaluated and approved this research protocol (Approval Number: 202005A026). The study adhered to the principles outlined in the Helsinki Declaration, and due to the retrospective nature of the data collection, the requirement for written informed consent was waived. All patient data were anonymized prior to analysis to ensure privacy protection. The studies were conducted in accordance with the local legislation and institutional requirements. Written informed consent for participation was not required from the participants or the participants” legal guardians/next of kin because this study is a retrospective analysis based on previously collected clinical data. All data were anonymized prior to analysis to ensure patient privacy and confidentiality. As no interventions were performed, and the study did not involve direct patient contact or influence on patient treatment, the Institutional Review Board (IRB) waived the requirement for written informed consent in accordance with ethical guidelines and regulations.

The correct ethics statement is “The studies involving humans were approved by the ethical review board of The First Hospital of Qinhuangdao, Qinhuangdao, Hebei, China, evaluated and approved this research protocol (Approval Number: 202401A111). The study adhered to the principles outlined in the Helsinki Declaration, and due to the retrospective nature of the data collection, the requirement for written informed consent was waived. All patient data were anonymized prior to analysis to ensure privacy protection. The studies were conducted in accordance with the local legislation and institutional requirements. Written informed consent for participation was not required from the participants or the participants' legal guardians/next of kin because this study is a retrospective analysis based on previously collected clinical data. All data were anonymized prior to analysis to ensure patient privacy and confidentiality. As no interventions were performed, and the study did not involve direct patient contact or influence on patient treatment, the Institutional Review Board (IRB) waived the requirement for written informed consent in accordance with ethical guidelines and regulations.”

The original version of this article has been updated.

